# Differential Regulation of Thyroid Hormone Metabolism Target Genes during Non-thyroidal Illness Syndrome Triggered by Fasting or Sepsis in Adult Mice

**DOI:** 10.3389/fphys.2017.00828

**Published:** 2017-10-25

**Authors:** Klaus N. Fontes, Adriana Cabanelas, Flavia F. Bloise, Cherley Borba Vieira de Andrade, Luana L. Souza, Marianna Wilieman, Isis H. Trevenzoli, Lais C. Agra, Johnatas D. Silva, Christianne Bandeira-Melo, Pedro L. Silva, Patricia R. M. Rocco, Tania M. Ortiga-Carvalho

**Affiliations:** ^1^Laboratory of Translational Endocrinology, Carlos Chagas Filho Institute of Biophysics, Federal University of Rio de Janeiro, Rio de Janeiro, Brazil; ^2^Laboratory of Molecular Endocrinology, Carlos Chagas Filho Institute of Biophysics, Federal University of Rio de Janeiro, Rio de Janeiro, Brazil; ^3^Laboratory of Inflammation, Carlos Chagas Filho Institute of Biophysics, Federal University of Rio de Janeiro, Rio de Janeiro, Brazil; ^4^Laboratory of Pulmonary Investigation, Carlos Chagas Filho Institute of Biophysics, Federal University of Rio de Janeiro, Rio de Janeiro, Brazil

**Keywords:** non-thyroidal illness syndrome, thyroid hormones, fasting, sepsis, CLP, thyroid hormone metabolism

## Abstract

Fasting and sepsis induce profound changes in thyroid hormone (TH) central and peripheral metabolism. These changes affect TH action and are called the non-thyroidal illness syndrome (NTIS). To date, it is still debated whether NTIS represents an adaptive response or a real hypothyroid state at the tissue level. Moreover, even though it has been considered the same syndrome, we hypothesized that fasting and sepsis induce a distinct set of changes in thyroid hormone metabolism. Herein, we aimed to evaluate the central and peripheral expression of genes involved in the transport (MCT8/*Slc16a2* and MCT10/*Slc16a10*), metabolism (*Dio1, Dio2*, and *Dio3*) and action (*Thra* and *Thrb*) of TH during NTIS induced by fasting or sepsis. Male mice were subjected to a 48 h period of fasting or cecal ligation and puncture (CLP)-induced sepsis. At the peripheral level, fasting led to: (1) reduced serum thyroxine (T_4_) and triiodothyronine (T_3_), expression of *Dio1, Thra, Slc16a2*, and MCT8 protein in liver; (2) increased hepatic Slc16a10 and Dio3 expression; and (3) decreased *Slc16a2* and *Slc16a10* expressions in the thyroid gland. Fasting resulted in reduction of *Tshb* expression in the pituitary and increased expression of Dio2 in total hypothalamus, arcuate (ARC) and paraventricular (PVN) nucleus. CLP induced sepsis resulted in reduced: (1) T_4_ serum levels; (2) *Dio1, Slc16a2, Slc16a10, Thra*, and *Thrb* expression in liver as well as *Slc16a2* expression in the thyroid gland (3) *Thrb* and *Tshb* mRNA expression in the pituitary; (4) total leukocyte counts in the bone marrow while increased its number in peritoneal and pleural fluids. In summary, fasting- or sepsis-driven NTIS promotes changes in the set point of hypothalamus-pituitary-thyroid axis through different mechanisms. Reduced hepatic THRs expression in conjunction with reduced TH transporters expression in the thyroid gland may indicate, respectively, reduction in the peripheral action and in the secretion of TH, which may contribute to the low TH serum levels observed in both models.

## Introduction

The non-thyroidal illness syndrome (NTIS), a clinical entity typically manifested by reduced conversion of thyroxine (T_4_) to triiodothyronine (T_3_) in different acute and chronic systemic conditions, is still a debated topic (Fliers et al., 2015). A long period of fasting induces a decrease in serum thyroid hormone levels (TH, T_4_, and T_3_) and an unresponsiveness in the hypothalamus-pituitary-thyroid (HPT) axis (Boelen et al., 2011; Fliers et al., 2014). Fasting also leads to a decrease in peripheral T_4_ to T_3_ conversion and reverse-T_3_ (rT_3_) clearance in humans (Warner and Beckett, 2010).

Sepsis can also lead to NTIS, with important consequences to the immunological system (van der Spek et al., 2017) and may affect critically ill patients outcome (Iervasi et al., 2003; Scoscia et al., 2004). During sepsis induced by LPS administration, TRH expression decreases through the up regulation of deiodinase type 2 in tanycytes, which further activates local T_4_, despite the low TH serum levels (Boelen et al., 2004b; De Vries et al., 2014).

It has long been postulated that abnormal levels of thyroid hormones in NTIS could represent an adaptive response to minimize metabolic demands during illness (Van den Berghe, 2014). However, it remains controversial whether NTIS is an adaptive response or a pathophysiological state that requires correction (Golombek, 2008). Additionally, serum thyroid hormone levels may not be indicative of tissue-specific T_3_ action because of the alterations in intracellular T_4_ and T_3_ uptake, local metabolism and receptor binding (Warner and Beckett, 2010).

The analysis of central and peripheral changes in thyroid hormone metabolism during NTIS allows a better understanding of systemic adaptive mechanisms that lead to HPT axis unresponsiveness during fasting and sepsis (Warner and Beckett, 2010; Boelen et al., 2011; Fliers et al., 2014). We hypothesized that sepsis and fasting would differentially change the expression of thyroid hormone metabolism target genes in HPT axis and liver of mice with more pronounced regulation in the Sepsis model. For this purpose, the objective of the present study is to evaluate, in an integrated view, the central and peripheral expression of genes involved in the transport (*Slc16a2* and *Slc16a10*), metabolism (*Dio1, Dio2*, and *Dio3*) and action (*Thra* and *Thrb*) of thyroid hormones in murine models of NTIS induced by fasting or cecal ligation puncture (CLP)-induced sepsis.

## Materials and methods

### Ethics statement

This study was approved by the Animal Care Committee of the Health Sciences Center, Federal University of Rio de Janeiro (CEUA-190/13), and registered with the Brazilian National Council for Animal Experimentation Control. All animals received humane care in compliance with the “Principles of Laboratory Animal Care” formulated by the National Society for Medical Research and the U.S. National Academy of Sciences Guide for the Care and Use of Laboratory Animals.

### Animal experiment

Male 129/SVxC57/BL6 mice, 10–12 weeks of age, weighting 22–25 g, were kept under a 12/12 h light/darkness cycle, in a controlled-temperature environment (23°C).

For fasting, in three independent experiments, 30 mice were divided into two groups (*n* = 15/group): one without access to food for 48 h (fasting group) and another group with food available *ad libitum* (control group). Both groups had free access to water. Twenty animals (*n* = 10/group) were used to evaluate mRNA expression and hormone levels. Ten animals (*n* = 5/group) were used to evaluate thyroid monocarboxilate transporter 8 by immunohistochemistry.

For sepsis, three independent experiments were performed. Sepsis was induced by cecal ligation and puncture surgery in 17 animals, whereas, 10 mice were *sham*-operated and used as control. Briefly, animals were anesthetized with intraperitoneal (i.p.) injection of ketamine and xylazine (0.25 and 0.025 mg/kg, respectively) and a midline laparotomy (2-cm incision) was performed. The cecum was carefully isolated to prevent damage to blood vessels and ligated with a 3-0 cotton suture. The ligature was placed below the ileocecal valve to prevent bowel obstruction. Finally, the cecum was punctured once with an 18-gauge needle and the animals left to recover from anesthesia (de Araujo et al., 2012). In *Sham* group, the abdominal cavity was opened and the cecum was isolated without ligation and puncture. Mice were fasted for 16 h before surgery in both groups. Postoperative care was similar in both groups, and consisted of a subcutaneous injection of tramadol hydrochloride (20 mg/g body weight) in 1 mL warm (37°C) normal saline (NaCl 0.9%). At 24 h, seven animals from CLP group died.

Mice were killed under anesthesia with isoflurane (Cristalia, Brazil) after 48 h of fasting or 24 h of sepsis induction, at 10:00 am in order to avoid hormonal and gene expression circadian variation. After killing, blood, thyroid, liver, hypothalamus, and pituitary were collected for molecular biology analysis (mRNA and protein expression by qPCR and Western Blot, respectively). Serum was obtained from the blood and stored at −20°C and all tissues were immediately frozen in liquid nitrogen and stored at −70°C. Bone marrow, peritoneal, and pleural fluids were collected for leukocyte analysis.

### Thyroid hormones serum measurement

Serum total T_3_ and T_4_ concentrations were measured by radioimmunoassay (RIA), using a commercial kit (Total T_4_ MAb RIA Kit and Total T_3_ RIA Kit, MP Biomedicals, CA, USA) and following the manufacturer's recommendations. This kit is based on solid phase method and the limit of detection for T_4_ was 1 μg/dL and for T_3_ was 25 ng/dL.

### mRNA extraction and real-time PCR reaction

Total RNA from liver, hypothalamus, pituitary, and thyroid samples was extracted using the TRIzol method, according to manufacturer's protocol (TRIzol Reagent; Life Technologies, CA, USA). In the fasting experiment, total RNA from PVN and ARC nuclei cryosections was extracted using the RNA easy microkit (Qiagen, Hilden, Germany) following manufacturer's recommendations. The cDNA synthesis was performed using the High Capacity cDNA Reverse Transcription Kit (Applied Biosystems, CA, USA) with 1 or 0.5 μg of total RNA, for tissue or nuclei cryosections respectively. All RNA samples were reverse transcribed in a single reaction. After the cDNA synthesis, mRNA expressions were evaluated by qPCR using the Maxima SYBR Green/ROX qPCR Master Mix 2X (Thermo Fisher Scientific, MA, USA) and the Master Cycler Realplex system (Eppendorf, Germany). Primer pair's sequences are shown in Table 1. The efficiency range accepted for each assay was 95–105%. qPCR quality and genomic DNA contamination was checked using intron-spanning primers, reverse transcriptase-negative samples from cDNA synthesis and melting curve analysis obtained from each reaction. Quantification of the samples mRNA expression was calculated from the quantification cycle (Cq) by the 2^−ΔΔCq^ method (Livak and Schmittgen, 2001) and corrected using 36β4 (*Rplp0*) expression as the reference gene. The results are expressed relative to the values of the control group (fasting) or *Sham* (CLP-induced sepsis), which were considered to be equal to 1.

**Table 1:** Primer's sequences used in RT-qPCR.

**Gene**	**Sequence**	**GenBank accession no**.
*Rplp0*	TGTTTGACAACGGCAGCATTT CCGAGGCAACAGTTGGGTA	NM_007475
*Slc16a2*	GAGCAGAGAGATTCCAGCAAGG GTAGGATGAGGGGTGGAGTGG	NM_009197
*Slc16a10*	TGTGGGATTCATTGGACTCATGT AGTGACGGCTGGTAGGCAAAG	NM_001114332
*Dio 1*	AGCCAGCTCTACGCGGC CCCTTGTAGCAGATCCTGCC	NM_007860.4
*Dio 2*	CATTCTGCTCAAGCACGTGGC GACGTGCACCACACTGGAATT	NM_010050.3
*Dio 3*	GTTTTTGGCTTGCTCTCAGG CAACAAGTCCGAGCTGTGAA	NM_172119.2
*Thra1*	CATCTTTGAACTGGGCAAGT CTGAGGCTTTAGACTTCCTGATC	NM_001313983
*Thrb1*	AACCAGTGCCAGGAATGTCG CTCTTCTCACGGTTCTCCTC	NM_001113417
*Tshb*	TCAACACCACCATCTGTGCT TTGCCACACTTGCAGCTTAC	NM_001165939
*Thrsp*	TGAGAACGACGCTGCTGAAAC AGGTGGGTAAGGATGTGATGGAG	NM_009381.3

*RT-qPCR, Real-time qPCR*.

### Hypothalamic paraventricular (PVN) and arcuate (ARC) nuclei microdissection

Hypothalamic PVN and ARC nuclei were obtained from snap frozen mouse brains (*n* = 10/group), previously collected with the other tissues, as described in “animal experiment” section. The nuclei were collected in cryosections by the punch technique, previously described (Franco et al., 2012, 2015), with particularities for the mouse brain, and following the mouse brain stereotaxic atlas coordinates (Paxinos and Franklin, 2004). The PVN and ARC nucleus were obtained through subsequently 640 and 1,500 μM slices. The slices were collected, respectively, at 0.58 mm and 1.22 mm posterior to the bregma.

### Western blotting

Liver samples were homogenized using the Tissuelyser LT (Qiagen, Hilden, Germany) with lyses buffer (pH 6.4; 50 mM HEPES, 1 mM MgCl2, 10 nM EDTA, and 1% Triton X) and a protease inhibitor cocktail (Roche/DSM Nutritional). Total protein extract (20 μg), previously denatured, was submitted to SDS-PAGE electrophoresis and transferred to a polyvinylidene difluoride membrane (PVDF Hybond-P; Amersham Biosciences, Buckinghamshire, UK). After that, the membrane was blocked for 2 h with 5% non-fat dry milk (Molico; Nestle, São Paulo, Brazil) and incubated overnight with anti-MCT8 antibody (1:750, Santa Cruz Biotechnology) and anti-cyclophilin antibody (1:250.000; Affinity Bioreagents, Golden, CO, USA), the loading control. Both primary antibodies were diluted in Tween-TBS (TBS + 0.1% Tween 20) with 2% non-fat dry milk. Membranes were then incubated for 3 h with secondary antibodies: HRP anti-goat IgG (1:7,000; Santa Cruz Biotechnology) and HRP anti-rabbit igG (1.2:10,000; Amersham Biosciences, Buckinghamshire, UK). After seven wash steps, the membranes were then incubated for chemiluminescence visualization with ECL (ECL Western Blotting System, Amershan Biosciences) and densitometry analysis was performed using the Image Quant Las4000 software (GE HealthCare Life Sciences, Buckinghamshire, UK). The results are expressed relative to the values of the control group (fasting) or *Sham* (CLP-induced sepsis), which were considered to be equal to 1.

### Immunohistochemistry

The thyroid glands were fixed in paraformaldehyde 4%. Cryopreservation was performed with sucrose gradient and posterior OCT embedding. A 5 μm cut was obtained with an ultramicrotome and the sections were then incubated with primary anti-MCT8 antibody (sc-47125, Santa Cruz Biotechnollogy), followed by the appropriate biotinylated secondary antibody (DCMT999, Spring), streptavidin-peroxidase (DHRR999, Spring) and finally 3,3-diamino-benzidine (DAB -ACB060, SCYTEK). The sections were then stained with Mayer's hematoxylin and mounted with glycerol. To quantify the MCT8 protein expression levels in the control and fasting groups, 30 random fields per animal were captured using an AXIOSCOPE microscope with an AXIOCAM HRc camera (CarlZeiss AG, Jena, Germany) at a magnification of 100× and analyzed with Image J analysis software.

### Total and differential leukocyte counts

In CLP induced sepsis animals, after 24 h, total and differential leukocyte counts were evaluated in bone marrow, peritoneal lavage fluid, and pleura. Peritoneal and pleural fluids were collected by flushing these cavities twice with 1.0 ml of 37°C sterile, PBS containing 10 mM EDTA using a micropippete. Mice femoral epiphysis were collected, centrifuged (98 g, 10 min, 4°C) and cell pellets were re-suspended in PBS for further quantification of leukocytes in the hemocytometer, as well as peritoneal and pleural washes, after dilution in Türk solution (1:40). After total leukocyte counts, samples from bone marrow and peritoneal/pleural washes were centrifuged (221 g, 10 min, 5°C). From the cell pellets obtained, differential cell counts were performed on May–Grunwald–Giemsa-stained cytospin preparations under an oil immersion objective to determine the percentage of mononuclear cells, eosinophils, and neutrophils in 100 leukocytes counted.

### Statistical analysis

Sample size calculation was based on previous experimental studies of fasting (de Vries et al., 2015) and sepsis (Bloise et al., 2016). A sample size of eight animals per group (allowing for one animal as dropout) would provide the appropriate power (1-β = 0.8) to identify significant (α = 0.05) differences in variable analyzed between injured (fasting or sepsis) and Control or *Sham* groups, taking into account an effect size d = 1.8, a two-sided test, and a sample size ratio = 1 (G^*^Power 3.1.9.2, University of Düsseldorf, Germany).

The Kolmogorov-Smirnov test with Lilliefors' corrections was used to test for normality of data, while the Levene median test was used to evaluate the homogeneity of variances. Rout test was used to remove outliers. The data are expressed as the mean ± the standard error of the mean (SEM). The Student's *t*-test was used for comparisons between two groups when data was normal otherwise Mann Whitney test was used. (mRNA expression, western blotting, RIA, and leukocyte counts). Statistical analyses were performed using the Graphpad prism 6 software (GraphPad Software, Inc., San Diego, CA, USA). Differences were considered to be significant at *P* < 0.05.

## Results

### Body weight and serum parameters

In fasting group, serum T_4_ levels were undetectable and serum T_3_ levels were lower (38%) in fasting mice compared to control (Table 2, *P* < 0.001). Fasting group also showed a 22% reduction in total body weight after a 48-h period of fasting (20.1 ± 1.4 g), compared to control (25.9 ± 0.9 g, *P* < 0.01).

**Table 2:** Serum T_4_ and T_3_ levels, in control and fasted mice (fasting) and sham and CLP-induced sepsis mice.

**Fasting**	**Control**	**Fasted**
T_4_ (μg/dL)	2.7 ± 0.3	ND
T_3_ (ng/dL)	80.1 ± 4.0	49.8 ± 4.6^***^
**CLP-induced sepsis**	***Sham***	**CLP**
T_4_ (μg/dL)	3.07 ± 0.03	1.82 ± 0.3^**^
T_3_ (ng/dL)	69.40 ± 5.6	57.52 ± 5.2

*T_4_, thyroxine, T_3_, triiodothyronine N = 9 animals/group. Values expressed as mean ± SEM*.

** P < 0.01;

*** *P < 0.001; ND, non-detectable*.

In CLP-induced sepsis group, serum T_4_ levels decreased 40% compared to *Sham* (Table 2, *P* < 0.01). No significant changes were observed in serum T_3_ levels between fasting and CLP groups.

### Total and differential leukocyte counts in CLP group

**Figure 1:**
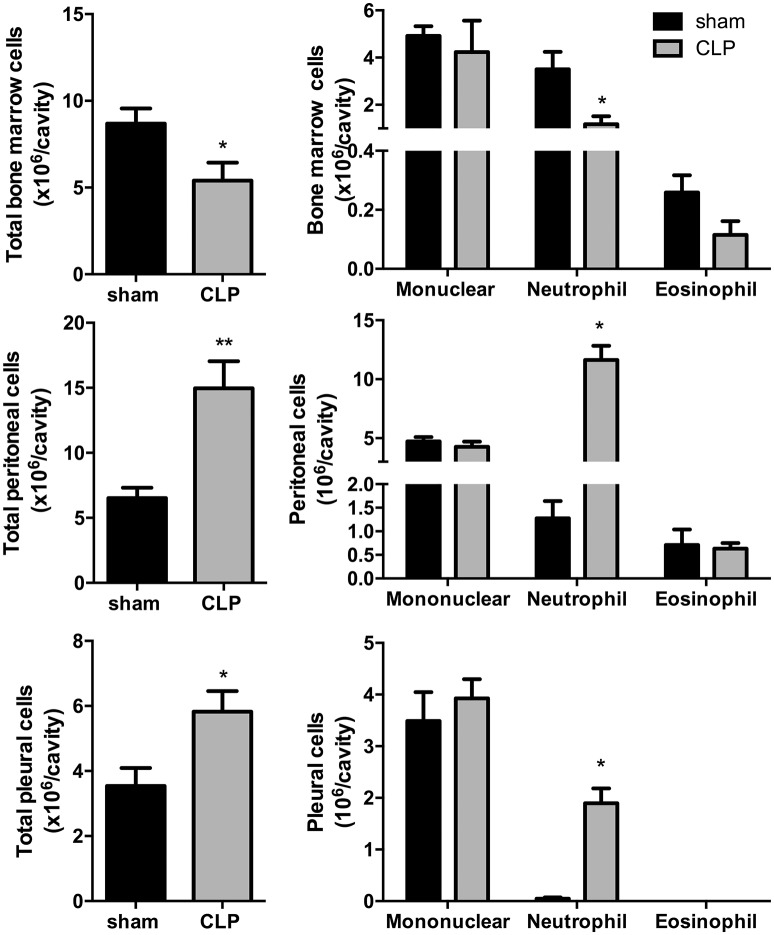
CLP-induced sepsis: total **(Left)** and differential **(Right)** leukocyte counts from bone marrow, peritoneal, and pleural lavage fluids. *N* = 8–9 animals/group. Values are expressed as mean±standard error of the mean (SEM). ^*^P < 0.05. ^**^P < 0.01.

Total leukocyte counts (Figure 1) in bone marrow decreased by 37% in CLP-induced sepsis group (*P* < 0.05) and increased by 2.5- and 1.5-fold, respectively, in the peritoneal and pleural lavages of the CLP group (*P* < 0.01 and *P* < 0.05, respectively). Differential leukocyte counts (Figure 1) showed a 67% decrease in bone marrow neutrophils of the CLP-induced sepsis group (*P* < 0.05). It was also observed an important increase in neutrophils of the peritoneal and pleural lavages in the CLP mice, compared to *Sham* group (*P* < 0.05).

### Liver thyroid hormone metabolism

**Figure 2:**
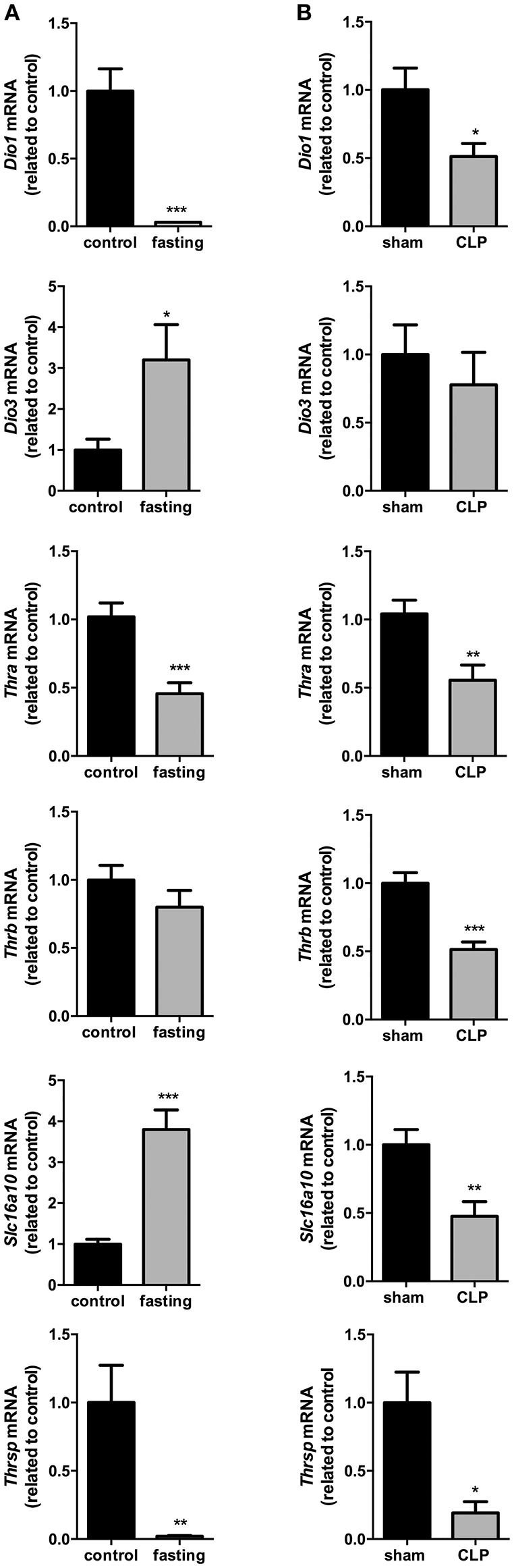
Thyroid hormone metabolism on fasted mice **(A)** and during CLP-induced sepsis **(B)**: *Dio1, Dio3, Thra, Thrb, Slc16a10*, and *Thrsp* mRNA expression in the liver. *N* = 6–9 animals/group. Values expressed as mean±SEM. ^*^P < 0.05. ^**^P < 0.01. ^***^P < 0.001.

The influence of fasting (Figure 2A) or CLP-induced sepsis (Figure 2B) on liver thyroid hormone metabolism was evaluated through the analysis of deiodinases mRNA expression. *Dio1* expression was decreased by 97% (*P* < 0.001) and *Dio3* expression had a 3-fold increase in fasting mice, compared to control group (*P* < 0.05). Since genomic actions of thyroid hormones depend on their binding to nuclear receptors (THRs), we evaluated the expression of *Thra* and *Thrb*, which encode to the transcription factors and T_3_ nuclear receptors Thrα and Thrβ, respectively. *Thra* expression decreased by 55% in fasting group compared to control (Figure 2A, *P* < 0.001), but *Thrb* expression did not change. To analyze whether the reduced *Thra* mRNA expression could reflect reduced T_3_ action, we investigated the mRNA expression of *Thrsp* in the liver, a T_3_ positive-regulated gene (Jump et al., 1984). Fasted mice showed a 98% reduction in *Thrsp* mRNA expression compared to control group (Figure 2A, *P* < 0.01).

In CLP-induced sepsis group, *Dio1* expression was decreased by 49% (Figure 2B, *P* < 0.05) and *Dio3* expression did not change between CLP-induced sepsis and *Sham* groups. CLP-induced sepsis mice showed a decrease in both *Thra* and *Thrb* mRNA expressions by 45% (*P* < 0.01) and 49% (*P* < 0.001), respectively, compared to *Sham* group. *CLP-induced sepsis* also promoted 87% decrease *Thrsp* mRNA expression in the liver (*P* < 0.05).

**Figure 3:**
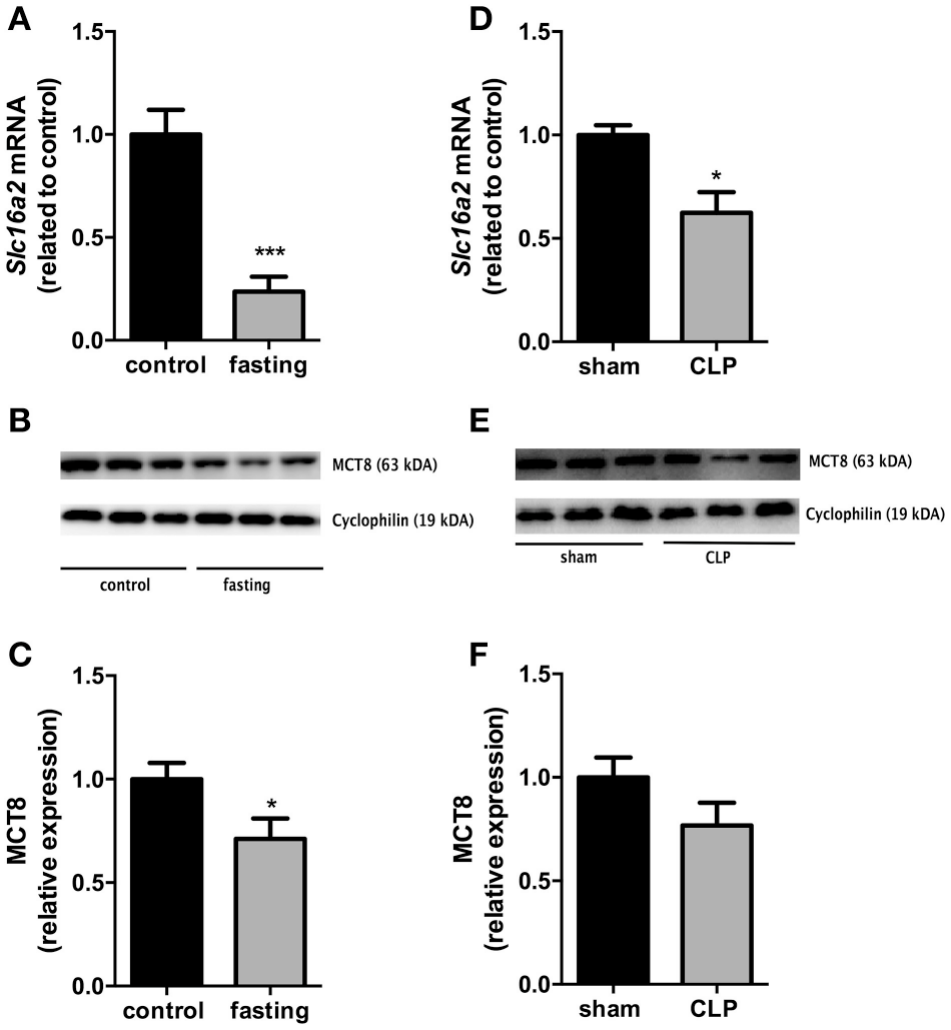
MCT8 expression on fasted mice **(A–C)** and during CLP-induced sepsis **(D–F)**: *Slc16a2* mRNA expression **(A,D)** and MCT8 protein expression in the liver **(B,C,E,F)**. *N* = 6 animals/group. Values expressed as mean ± SEM. ^*^P < 0.05. ^***^P < 0.001.

We then investigated the expression of the monocarboxylate transporters MCT8 and MCT10. Fasting promoted a 4-fold increase in *Slc16a10* mRNA expression (Figure 2A, *P* < 0.001) and, in contrast, decreased by 77% *Slc16a2* gene expression (Figure 3A, *P* < 0.001). Furthermore, fasted mice showed a 30% reduction in MCT8 protein expression in the liver compared to the control group (Figures 3B,C, *P* < 0.05). In contrast, CLP-induced sepsis promoted a 53% decrease in liver *Slc16a10* mRNA expression (Figure 2B, *P* < 0.01) and a 49% decrease in liver *Slc16a*2 mRNA expression (Figure 3D, *P* < 0.05). No differences were seen on MCT8 protein expression in CLP-induced sepsis (Figures 3E,F).

### Expression of monocarboxilate transporters in thyroid

**Figure 4:**
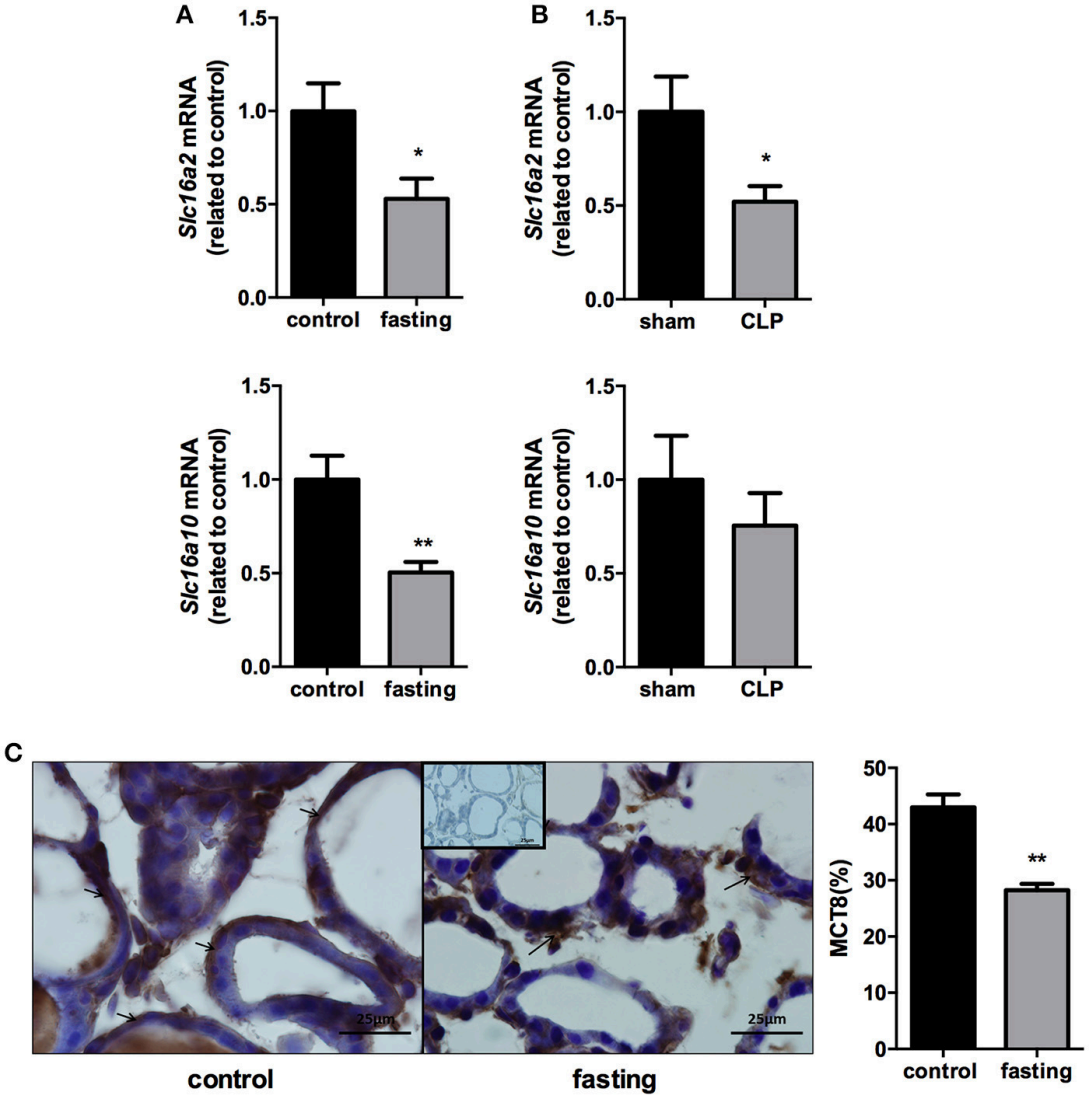
*Slc16a2* and *Slc16a10* mRNA expression in the thyroid of fasted **(A)** and CLP-induced sepsis **(B)**. Photomicrography and relative quantification of MCT8 immunostaining in the thyroid gland on fasted mice **(C)**. The black arrows show MCT8 staining in the thyrocytes basolateral membranes. Inset represents the negative control. *N* = 9 animals/group **(A,B)** and five animals/group **(C)**. Values expressed as mean±SEM. ^*^P < 0.05. ^**^P < 0.01.

We analyzed the expression of monocarboxilate transporters (MCT8 and MCT10) in the thyroid gland to investigate a possible role of this gland, during fasting and CLP-induced sepsis, causing changes in serum thyroid hormone levels. mRNA expression of *Slc16a2* and *Slc16a10* was decreased by 50% in fasting mice, compared to control group (Figure 4A, *P* < 0.05 and *P* < 0.01, respectively). In CLP-induced sepsis, *Slc16a2* mRNA expression decreased 48% compared to control (Figure 4B, *P* < 0.05). *Slc16a10* mRNA expression did not differ between *Sham* and CLP-induced sepsis (Figure 4B). Furthermore, immunohistochemistry analysis showed that fasting decreased MCT8 staining in the thyrocytes (Figure 4C, *P* < 0.01).

### Central thyroid hormone metabolism

Since NTIS changes the set point of HPT axis through different mechanisms in fasting or CLP-induced sepsis, we set out to evaluate the expression of genes involved in thyroid hormone metabolism in the pituitary and the hypothalamus.

**Figure 5:**
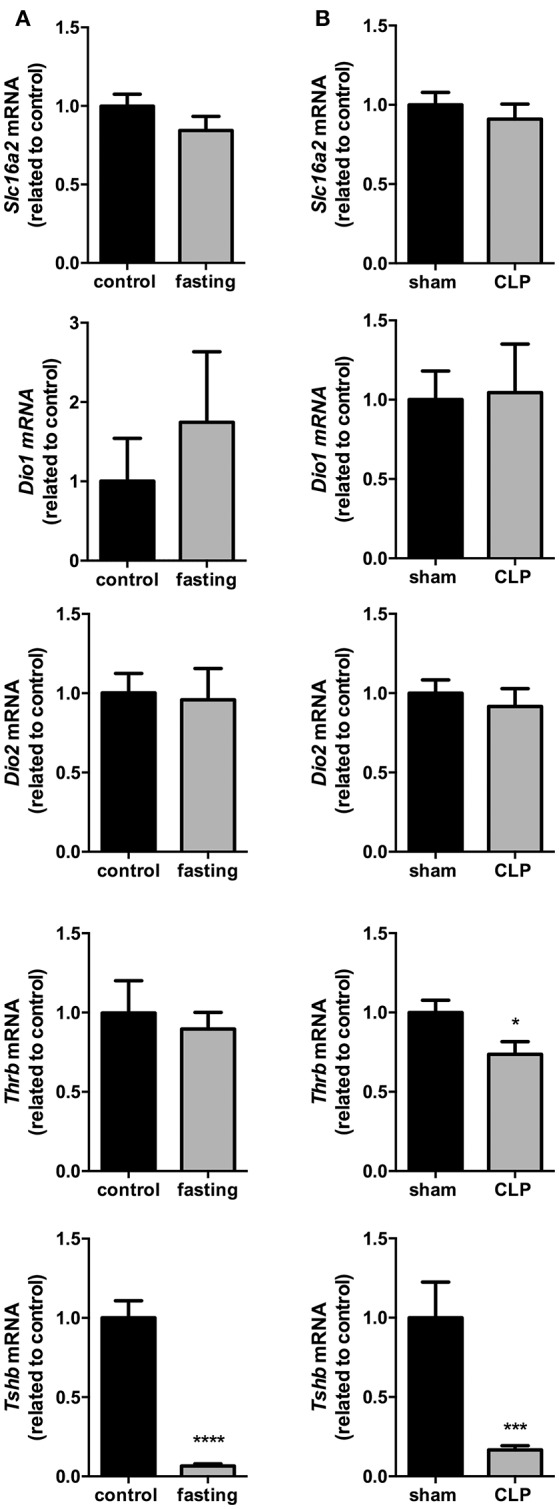
Pituitary thyroid hormones metabolism on fasted mice **(A)** and during CLP-induced sepsis **(B)**. *Slc16a2, Dio1, Dio2, Thrb*, and *Tshb* mRNA expression in the pituitary. *N* = 6–9 animals/group. Values expressed as mean±SEM. ^*^P < 0.05. ^***^P < 0.001. ^****^P < 0.0001.

The expression of *Slc16a2, Dio1, Dio2*, and *Thrb* in the pituitary did no change in fasting mice (Figure 5A), although fasting promoted a 94% reduction in *Tshb* mRNA expression (Figure 5A, *P* < 0.0001), which suggest a decrease in the response of the pituitary to the low serum levels of T_4_.

*Slc16a2, Dio1*, and *Dio2* mRNA expressions did not differ between *Sham* and CLP-induced sepsis groups (Figure 5B). However, both *Thrb* and *Tshb* mRNA expressions reduced 27% (Figure 5B, *P* < 0.05) and 76% (Figure 5B, *P* < 0.001), respectively, confirming that in this model of NTIS, HPT axis is also less responsive to low T_4_ serum levels.

**Figure 6:**
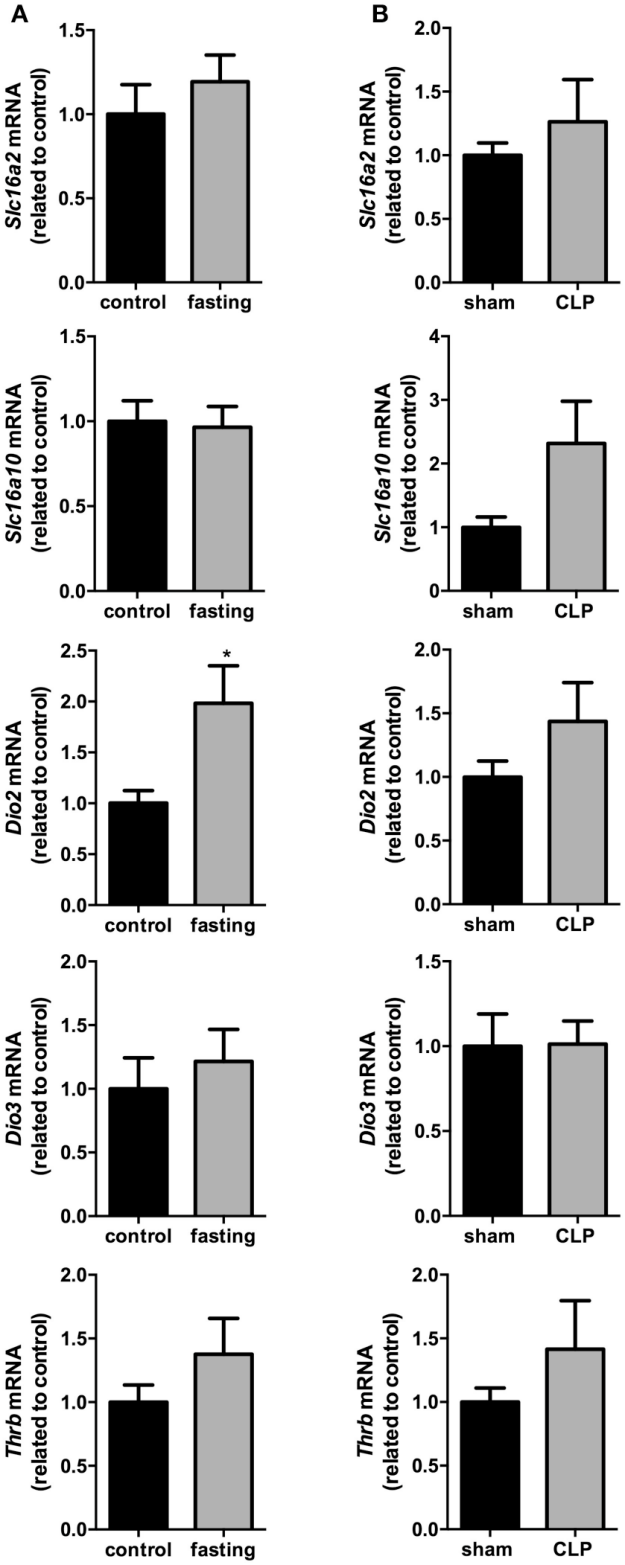
Hypothalamus thyroid hormones metabolism on fasted mice **(A)** and during CLP-induced sepsis **(B)**. *Slc16a2, Slc16a10, Dio2, Dio3*, and *Thrb* mRNA expression in the hypothalamus. *N* = 6–8 animals/group. Values expressed as mean±SEM. ^*^P < 0.05.

In the total hypothalamus, the expression of *Slc16a2, Slc16a10, Dio3*, and *Thrb* did not change in these two models fasting and CLP-induced sepsis (Figures 6A,B). However, we observed a 2-fold increase in the expression of *Dio2* mRNA in fasting mice, compared to control group (Figure 6A, *P* < 0.05). *Dio2* expression did not change in the total hypothalamus of CLP-induced sepsis mice, compared to control group (Figure 6B). Thus, we decided to perform a more thorough analysis of *Dio2* expression in PVN and ARC nuclei only on the fasting model.

### Expression of *Dio2* in the PVN and ARC nucleus (fasting experiment)

**Figure 7:**
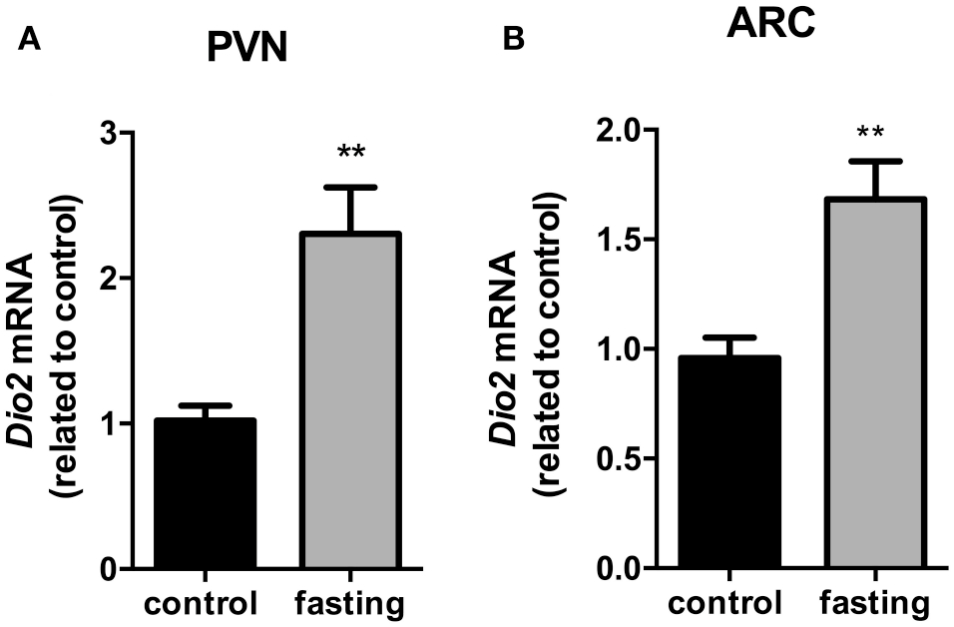
Dio2 mRNA expression in the hypothalamic PVN **(A)** and ARC **(B)** nucleus on fasted mice. PVN, paraventricular nucleus. ARC, arcuate nucleus. *N* = 8 animals/groups. Values expressed as mean±SEM.^**^P < 0.01.

We then investigated the *Dio2* expression in the PVN and ARC nucleus. In the PVN, *Dio2* mRNA expression presented a 2.3-fold increase in fasting group (Figure 7, *P* < 0.01). In the ARC, fasting promoted a 1.6-fold increase in *Dio2* mRNA expression (Figure 7, *P* < 0.01).

## Discussion

In the present study we showed that fasting and CLP-induced sepsis promoted changes in the systemic TH economy through different mechanisms. Our main finding indicates that, during fasting, *Dio2* up-regulation in PVN/ARC nuclei seems to be an adaptive mechanism involved in the HPT-axis impairment, while in CLP-induced sepsis this impairment is probably associated with inflammatory mediators and independent of T_3_ uptake and action.

CLP model for induction of sepsis in mice is considered the gold standard for sepsis research, inducing a polymicrobial sepsis and mimicking the inflammatory and vascular patterns observed in humans (Dejager et al., 2011). However, CLP-induced sepsis is still an underexplored murine model of NTIS (Dejager et al., 2011). In our study, we observed a decrease in *Dio1* liver expression of CLP-induced septic mice, which is considered a hallmark of NTIS according to previous studies (O'Mara et al., 1993; Boelen et al., 1996; de Vries et al., 2015). This finding is related to the influence of inflammatory cytokines on THRβ and THRα expression (Boelen et al., 2004a; Kwakkel et al., 2010) and the redox state of the hepatocytes (Wajner et al., 2011). The reduction in the neutrophils counts in bone marrow and an increase in peritoneal and pleural lavages in the CLP-induced sepsis group confirmed what is observed in abdominal septic patients (Asaduzzaman et al., 2008). We also observed a decrease in serum T_4_ levels after 24 h of CLP surgery another hallmarks of NTIS in mice, confirming the CLP-induced sepsis as a model for NTIS.

Fasting promotes profound changes in thyroid hormone economy and its effects on central and peripheral TH metabolism in rodents are similar to those observed in humans (Boelen et al., 2008), which makes it an efficient and easy-to-execute experimental model for understanding similarities and differences that may arise from the inflammatory stimulus *per se* or from the food deprivation state. A recent study from Galton et al. demonstrated that the decrease in serum T_3_ and T_4_ levels during fasting occurs even in the absence of deiodinase type I. However, D3 knockout (D3KO) mice demonstrate a blunting of the fasting-induced decrease in serum T_4_ and T_3_ levels, revealing that D3 is, in part, responsible for the adaptive systemic changes in thyroid hormone economy during food deprivation (Galton et al., 2014). In contrast to the increase in *Dio3* expression during fasting, we did not observe changes in *Dio3* expression in the CLP-induced sepsis. However, thyroid hormone serum levels in D3KO mice subjected to bacterial sepsis decreased equally compared to WT mice (Boelen et al., 2009), which indicates that D3 does not play an important role in the decrease in thyroid hormone levels during sepsis.

In both models, we observed decreased TH serum levels and hepatic *Dio1* mRNA, which could be caused by a decrease in TH influx into the hepatocytes, therefore we investigated the expression of the two main TH transporters in the liver, MCT8 and MCT10. In both models, MCT8 expression was decreased what suggests, together with hepatic deiodinases decreased part of a particular adaptive mechanism to reduce energy expenditure. In contrast to our results, a recent study in a rabbit model of prolonged critical illness demonstrated an increase in the hepatic expression of *Slc16a2*, which was inversely correlated with serum thyroid hormone parameters (Mebis et al., 2009). The differences between immunological effectors in acute and prolonged stage of critical illness (Van den Berghe, 2014) and also in the induction of NTIS can be the explanation of such down regulation.

While MCT8 is a specific T_3_ and T_4_ transporter, MCT10 transports not only thyroid hormones but also aromatic amino acids (Van Der Deure et al., 2010). In a recent study, de Vries et al. described an increase in *Slc16a10* expression after 36 h of fasting in male rats (de Vries et al., 2015). Our results in our mice fasting model, corroborate de Vries' results. However, we did not observe changes in *Slc16a10* expression in the liver of CLP-induced sepsis mice. The increase in MCT10 expression in fasting may affect thyroid hormone transport but also can represent a compensatory response to the intense protein catabolism during fasting.

Once we observed a reduction in hepatic *Slc16a2* expression in both models, we hypothesized that T_3_ content in the liver could also be reduced. Then, we investigated the expression of both TH receptors isoforms (*Thra* and *Thrb*) and a classic TH up-regulated gene, *Thrsp* (Jump et al., 1984). While *Thrb* expression did not change after fasting, *Thra* expression decreased by half, fact that can explain, in part, the reduction in thyroid hormone action, confirmed by the decrease in liver *Thrsp* expression in fasted mice. However, once ~60% of liver T_3_-regulated genes are THRβ-dependent (Boelen et al., 2011), and *Dio1* (THRβ-mainly regulated gene) expression was reduced in the liver, we speculate that, in fact, liver T_3_ content is reduced. In the CLP-induced sepsis, the reduction in both *Thra* and *Thrb* mRNA and in *Thrsp* mRNA expression confirms a decrease in T_3_ action in liver, in agreement with the literature (Beigneux et al., 2003).

The function of MCT8 in the thyroid gland during NITS models has been unexplored in the literature. Different from other tissues, in the thyroid, MCT8 is involved in the secretion of T_4_ and T_3_ (Di Cosmo et al., 2010). We observed a decrease in *Slc16a2* expression in both fasting and CLP-sepsis models. Thus, we suggest that the decrease in the expression of TH transporters in the thyroid gland is contributing to low levels of serum TH levels in both models. Altogether, we hypothesize that thyroid has a major role in the systemic thyroid economy during NTIS, with a notable contribution to the low serum T_4_ levels observed in both experimental models.

CLP-induced sepsis lead to a paradoxical decrease in both *Tshb* and *Thrb* mRNA expressions in the pituitary. Although THRβ mainly exerts the negative feedback regulation of the HPT axis, *in vitro* and *in vivo* studies show that inflammatory cytokines can regulate independently both genes during acute inflammation (Wassen et al., 1996; Boelen et al., 2004b). Then, we hypothesize that the impairment of HPT-axis during sepsis is due to the effects of inflammatory mediators and occurs independently of T_3_ uptake and action.

Coppola et al. in a very elegant study, revealed a role for hypothalamic D2, which leads to increased local T_3_ production, triggering the expression of uncoupling protein 2 (UCP2) and the activation of NPY/AgRP neurons in the arcuate nucleus during fasting (Coppola et al., 2007). In attempt to investigate the dramatic reduction in *Tshb* expression, and since *Dio2* expression did not change in the total hypothalamus of CLP-induced sepsis mice, we decided to perform a more thorough analysis of *Dio2* expression in PVN and ARC nuclei only during fasting. We observed an increase of *Dio2* gene expression in total hypothalamus and in both PVN and ARC nucleus of fasted mice. It is consistent to conclude that the impairment in HPT axis during fasting is a consequence of the local hypothalamic hyperthyroid state, which in turn leads to the inappropriate *Tshb* reduced mRNA expression in the pituitary.

Finally, our NTIS models, promotes changes in thyroid hormone metabolism and HPT axis that could be part of a systemic adaptive response to reduce the energy expenditure in both models. At the peripheral level, fasting and sepsis promotes changes in the expression of thyroid hormone transporters and deiodinases in the liver, suggesting a decrease in T_4_ activation, along with changes in the expression of thyroid hormone transporters in the thyroid, which contribute to reduced thyroid hormone serum levels. At the central level, we suggest a role for the PVN and ARC in the generation of a hypothalamic hyperthyroid state during fasting, one of the NTIS hallmarks. In the septic pituitary, the decrease in *Tshb* and *Thrb* expressions, explains the impairment of the HPT axis independently of T_3_ uptake and action.

## Conclusion

Although fasting and CLP-induced sepsis caused similar changes in peripheral thyroid hormone economy, the profound changes in the set point of HPT axis that characterize NITS are caused through different mechanisms.

## Author contributions

Conceptualization of the experiments: KF, AC, FB, CA, LS, LA, CB, PS, PR, and TO. Formal analysis: KF, AC, FB, CA, LS, MW, IT, LA, JS, CB, PS, PR, and TO. Funding acquisition: TO. Performed experiments: KF, AC, FB, CA, LS, MW, LA, JS, and PS. Project supervision: FB, CB, PS, PR, and TO. Writing draft: KF, AC, FB, CA, LS, MW, IT, LA, JS, CB, PS, PR, and TO. Writing review and editing: KF, AC, FB, LS, IT, CB, PS, PR, and TO.

### Conflict of interest statement

The authors declare that the research was conducted in the absence of any commercial or financial relationships that could be construed as a potential conflict of interest.

## References

[B1] Asaduzzaman M. Zhang S. Lavasani S. Wang Y. Thorlacius H. (2008). LFA-1 and MAC-1 mediate pulmonary recruitment of neutrophils and tissue damage in abdominal sepsis. Shock 30, 254–259. 10.1097/shk.0b013e318162c567 18197144

[B2] Beigneux A. Moser A. Shigenaga J. Grunfeld C. Feingold K. (2003). Sick euthyroid syndrome is associated with decreased TR expression and DNA binding in mouse liver. Am. J. Physiol. Endocrinol. Metab. 284, E228–E236. 10.1152/ajpendo.00155.2002 12388159

[B3] Bloise F. F. van der Spek A. H. Surovtseva O. V. Ortiga-Carvalho T. M. Fliers E. Boelen A. (2016). Differential effects of sepsis and chronic inflammation on diaphragm muscle fiber type, thyroid hormone metabolism, and mitochondrial function. Thyroid 26, 600–609. 10.1089/thy.2015.0536 26892873

[B4] Boelen A. Kwakkel J. Fliers E. (2011). Beyond low plasma T3: local thyroid hormone metabolism during inflammation and infection. Endocr. Rev. 32, 670–693. 10.1210/er.2011-0007 21791567

[B5] Boelen A. Kwakkel J. Platvoet-ter Schiphorst M. Mentrup B. Baur A. Koehrle J.. (2004a). Interleukin-18, a proinflammatory cytokine, contributes to the pathogenesis of non-thyroidal illness mainly via the central part of the hypothalamus-pituitary-thyroid axis. Eur. J. Endocrinol. 151, 497–502. 10.1530/eje.0.1510497 15476451

[B6] Boelen A. Kwakkel J. Thijssen-Timmer D. C. Alkemade A. Fliers E. Wiersinga W. M. (2004b). Simultaneous changes in central and peripheral components of the hypothalamus-pituitary-thyroid axis in lipopolysaccharide-induced acute illness in mice. J. Endocrinol. 182, 315–323. 10.1677/joe.0.1820315 15283692

[B7] Boelen A. Kwakkel J. Wieland C. W. St Germain D. L. Fliers E. Hernandez A. (2009). Impaired bacterial clearance in type 3 deiodinase-deficient mice infected with *Streptococcus pneumoniae*. Endocrinology 150, 1984–1990. 10.1210/en.2008-1133 19036878 PMC2659279

[B8] Boelen A. Platvoet-ter Schiphorst M. C. Van Rooijen N. Wiersinga W. M. (1996). Selective macrophage depletion in the liver does not prevent the development of the sick euthyroid syndrome in the mouse. Eur. J. Endocrinol. 134, 513–518. 8640306 10.1530/eje.0.1340513

[B9] Boelen A. Wiersinga W. M. Fliers E. (2008). Fasting-induced changes in the hypothalamus-pituitary-thyroid axis. Thyroid 18, 123–129. 10.1089/thy.2007.0253 18225975

[B10] Coppola A. Liu Z. W. Andrews Z. B. Paradis E. Roy M. C. Friedman J. M.. (2007). A central thermogenic-like mechanism in feeding regulation: an interplay between arcuate nucleus T3 and UCP2. Cell Metab. 5, 21–33. 10.1016/j.cmet.2006.12.002 17189204 PMC1783766

[B11] de Araujo C. C. Silva J. D. Samary C. S. Guimaraes I. H. Marques P. S. Oliveira G. P.. (2012). Regular and moderate exercise before experimental sepsis reduces the risk of lung and distal organ injury. J. Appl. Physiol. 112, 1206–1214. 10.1152/japplphysiol.01061.2011 22267391

[B12] Dejager L. Pinheiro I. Dejonckheere E. Libert C. (2011). Cecal ligation and puncture: the gold standard model for polymicrobial sepsis? Trends Microbiol. 19, 198–208. 10.1016/j.tim.2011.01.001 21296575

[B13] De Vries E. M. Kwakkel J. Eggels L. Kalsbeek A. Barrett P. Fliers E.. (2014). NFκB signaling is essential for the lipopolysaccharide-induced increase of type 2 deiodinase in tanycytes. Endocrinology 155, 2000–2008. 10.1210/en.2013-2018 24635351

[B14] de Vries E. M. van Beeren H. C. Ackermans M. T. Kalsbeek A. Fliers E. Boelen A. (2015). Differential effects of fasting vs food restriction on liver thyroid hormone metabolism in male rats. J. Endocrinol. 224, 25–35. 10.1530/JOE-14-0533 25349245

[B15] Di Cosmo C. Liao X. H. Dumitrescu A. M. Philp N. J. Weiss R. E. Refetoff S. (2010). Mice deficient in MCT8 reveal a mechanism regulating thyroid hormone secretion. J. Clin. Invest. 120, 3377–3388. 10.1172/JCI42113 20679730 PMC2929715

[B16] Fliers E. Bianco A. C. Langouche L. Boelen A. (2015). Thyroid function in critically ill patients. Lancet. Diabetes Endocrinol. 3, 816–825. 10.1016/S2213-8587(15)00225-9 26071885 PMC4979220

[B17] Fliers E. Kalsbeek A. Boelen A. (2014). Mechanisms in endocrinology: beyond the fixed setpoint of the hypothalamus-pituitary-thyroid axis. Eur. J. Endocrinol. 171, R197–R208. 10.1530/EJE-14-0285 25005935

[B18] Franco J. G. Dias-Rocha C. P. Fernandes T. P. Albuquerque Maia L. Lisboa P. C. Moura E. G.. (2015). Resveratrol treatment rescues hyperleptinemia and improves hypothalamic leptin signaling programmed by maternal high-fat diet in rats. Eur. J. Nutr. 55, 601–610. 10.1007/s00394-015-0880-7 25801629

[B19] Franco J. G. Fernandes T. P. Rocha C. P. D. Calviño C. Pazos-Moura C. C. Lisboa P. C.. (2012). Maternal high-fat diet induces obesity and adrenal and thyroid dysfunction in male rat offspring at weaning. J. Physiol. 590, 5503–5518. 10.1113/jphysiol.2012.240655 22869015 PMC3515834

[B20] Galton V. A. Hernandez A. St. Germain D. L. (2014). The 5-deiodinases are not essential for the fasting-induced decrease in circulating thyroid hormone levels in male mice: possible roles for the type 3 deiodinase and tissue sequestration of hormone. Endocrinology 155, 3172–3181. 10.1210/en.2013-1884 24635350 PMC4097997

[B21] Golombek S. G. (2008). Nonthyroidal illness syndrome and euthyroid sick syndrome in intensive care patients. Semin. Perinatol. 32, 413–418. 10.1053/j.semperi.2008.09.010 19007679

[B22] Iervasi G. Pingitore A. Landi P. Raciti M. Ripoli A. Scarlattini M.. (2003). Low-T3 syndrome: a strong prognostic predictor of death in patients with heart disease. Circulation 107, 708–713. 10.1161/01.CIR.0000048124.64204.3F 12578873

[B23] Jump D. B. Narayan P. Towle H. Oppenheimer J. H. (1984). Rapid effects of triiodothyronine on hepatic gene expression. Hybridization analysis of tissue-specific triiodothyronine regulation of mRNA(S14). J. Biol. Chem. 259, 2789–2797. 6199351

[B24] Kwakkel J. Chassande O. van Beeren H. C. Fliers E. Wiersinga W. M. Boelen A. (2010). Thyroid hormone receptor α modulates lipopolysaccharide-induced changes in peripheral thyroid hormone metabolism. Endocrinology 151, 1959–1969. 10.1210/en.2009-1049 20194731

[B25] Livak K. J. Schmittgen T. D. (2001). Analysis of relative gene expression data using real-time quantitative PCR and the 2^-ΔΔC^_T_ Method. Methods 25, 402–408. 10.1006/meth.2001.1262 11846609

[B26] Mebis L. Paletta D. Debaveye Y. Ellger B. Langouche L. D'Hoore A.. (2009). Expression of thyroid hormone transporters during critical illness. Eur. J. Endocrinol. 161, 243–250. 10.1530/EJE-09-0290 19439506

[B27] O'Mara B. A. Dittrich W. Lauterio T. J. St. Germain D. L. (1993). Pretranslational regulation of type I 5′-deiodinase by thyroid hormones and in fasted and diabetic rats. Endocrinology 133, 1715–1723. 8404614 10.1210/endo.133.4.8404614

[B28] Paxinos G. Franklin K. B. J. (2004). The mouse brain in stereotaxic coordinates. Psychoneuroendocrinology 28, 827–828. 10.1016/S0306-4530(03)00088-X

[B29] Scoscia E. Baglioni S. Eslami A. Iervasi G. Monti S. Todisco T. (2004). Low triiodothyronine (T3) state: a predictor of outcome in respiratory failure? Results of a clinical pilot study. Eur. J. Endocrinol. 151, 557–560. 10.1530/eje.0.1510557 15538932

[B30] Van den Berghe G. (2014). Non-thyroidal illness in the ICU: a syndrome with different faces. Thyroid 24, 1456–1465. 10.1089/thy.2014.0201 24845024 PMC4195234

[B31] Van Der Deure W. M. Peeters R. P. Visser T. J. (2010). Molecular aspects of thyroid hormone transporters, including MCT8, MCT10, and OATPs, and the effects of genetic variation in these transporters. J. Mol. Endocrinol. 44, 1–11. 10.1677/JME-09-0042 19541799

[B32] van der Spek A. H. Fliers E. Boelen A. (2017). Thyroid hormone metabolism in innate immune cells. J. Endocrinol. 232, R67–R81. 10.1530/JOE-16-0462 27852725

[B33] Wajner S. M. Goemann I. M. Bueno A. L. Larsen P. R. Maia A. L. (2011). IL-6 promotes nonthyroidal illness syndrome by blocking thyroxine activation while promoting thyroid hormone inactivation in human cells. J. Clin. Invest. 121, 1834–1845. 10.1172/JCI44678 21540553 PMC3083773

[B34] Warner M. H. Beckett G. J. (2010). Mechanisms behind the non-thyroidal illness syndrome: an update. J. Endocrinol. 205, 1–13. 10.1677/JOE-09-0412 20016054

[B35] Wassen F. W. Moerings E. P. Van Toor H. De Vrey E. A. Hennemann G. Everts M. E. (1996). Effects of interleukin-1 beta on thyrotropin secretion and thyroid hormone uptake in cultured rat anterior pituitary cells. Endocrinology 137, 1591–1598. 8612490 10.1210/endo.137.5.8612490

