# Corrigendum: Differential Regulation of Thyroid Hormone Metabolism Target Genes during Non-thyroidal Illness Syndrome Triggered by Fasting or Sepsis in Adult Mice

**DOI:** 10.3389/fphys.2017.00995

**Published:** 2017-11-28

**Authors:** Klaus N. Fontes, Adriana Cabanelas, Flavia F. Bloise, Cherley Borba Vieira de Andrade, Luana L. Souza, Marianna Wilieman, Isis H. Trevenzoli, Lais C. Agra, Johnatas D. Silva, Christianne Bandeira-Melo, Pedro L. Silva, Patricia R. M. Rocco, Tania M. Ortiga-Carvalho

**Affiliations:** ^1^Laboratory of Translational Endocrinology, Carlos Chagas Filho Institute of Biophysics, Federal University of Rio de Janeiro, Rio de Janeiro, Brazil; ^2^Laboratory of Molecular Endocrinology, Carlos Chagas Filho Institute of Biophysics, Federal University of Rio de Janeiro, Rio de Janeiro, Brazil; ^3^Laboratory of Inflammation, Carlos Chagas Filho Institute of Biophysics, Federal University of Rio de Janeiro, Rio de Janeiro, Brazil; ^4^Laboratory of Pulmonary Investigation, Carlos Chagas Filho Institute of Biophysics, Federal University of Rio de Janeiro, Rio de Janeiro, Brazil

**Keywords:** non-thyroidal illness syndrome, thyroid hormones, fasting, sepsis, CLP, thyroid hormone metabolism

In the original article, there was an error. Differential Regulation of Thyroid Hormone Metabolism Target Genes during Non-thyoidal Illness Syndrome Triggered by Fasting or Sepsis in Adult Mice.

Correction has been made to Article title.

Differential Regulation of Thyroid Hormone Metabolism Target Genes during Non-thyroidal Illness Syndrome Triggered by Fasting or Sepsis in Adult Mice

The authors apologize for this error and state that this does not change the scientific conclusions of the article in any way.

The original article has been updated.

## Conflict of interest statement

The authors declare that the research was conducted in the absence of any commercial or financial relationships that could be construed as a potential conflict of interest.

